# Ultrasound-Guided Ethanol Ablation for Thyroglossal Duct Cyst: A Review of Technical Issues and Potential as a New Standard Treatment

**DOI:** 10.3390/jcm12175445

**Published:** 2023-08-22

**Authors:** Dongbin Ahn

**Affiliations:** Department of Otolaryngology-Head and Neck Surgery, School of Medicine, Kyungpook National University, 130 Dongdeok-ro, Jung-gu, Daegu 41944, Republic of Korea; godlikeu@naver.com; Tel.: +82-53-200-5781; Fax: +82-53-423-4524

**Keywords:** thyroglossal duct cyst, ultrasound, ethanol, ablation, Sistrunk, sclerotherapy

## Abstract

The thyroglossal duct cyst (TGDC) is the most common congenital neck mass, accounting for 70–75% of all congenital neck masses. Although the Sistrunk operation has been used as a standard of treatment, it is accompanied by a considerable surgical burden, including the need for general anesthesia, a visible surgical scar on the neck surface, and postoperative complications. Ultrasound-guided ethanol ablation (US-EA) is a minimally invasive and office-based technique that is widely used as a non-surgical treatment for several benign cystic lesions, particularly benign thyroid cysts. Recently, US-EA has also been gaining popularity as a good alternative for TGDC treatment, which is associated with high feasibility, a high safety profile, and favorable treatment outcomes. To our best knowledge, seven studies on the use of EA as a primary treatment option for TGDC have been published since 2011. Although these studies have reported promising results, there is a lack of consensus on several issues regarding the application of EA for TGDC, particularly its detailed techniques and role as a primary treatment. This article aims to provide a comprehensive review of EA for TGDC, addressing technical issues and its possible role as a new standard of treatment for TGDC.

## 1. Introduction

The thyroglossal duct cyst (TGDC) is the most common congenital neck mass and a remnant of the thyroid anlage during embryonic development of the thyroid gland, representing more than 70–75% of congenital neck masses [1,2,3,4,5]. Although TGDC is benign in most cases and may have minimal clinical significance, treatment is usually recommended based on various reasons, such as cosmetic issues, recurrent infections, and concerns of occult malignancy [2,6,7,8].

Most otolaryngology textbooks and studies on TGDC elucidate that the treatment of TGDC is primarily surgical, while other treatment options are rarely suggested [1,2,4,9]. In terms of surgical treatment, the Sistrunk procedure, which involves the complete excision of the TGDC and a central portion of the hyoid bone, has been considered the surgery of choice since its first introduction in 1920 [1,4]. Although this procedure remarkably contributed to the successful management of TGDC by reducing the recurrence rate to 1–5% from 45–55% after simple excision, it entails various surgical burdens, including the need for general anesthesia, a visible surgical scar on the neck surface, and possible postoperative complications [1,3,4,7,10]. Particularly, given that the most common clinical presentation of TGDC is a painless neck mass, the most common rationale for TGDC treatment is the resolution of the cosmetic deformity caused by the TGDC mass; surgery may not be a patient-oriented treatment as it usually leaves postoperative surgical scars, defeating the purpose of resolving the cosmetic problems as a result of the TGDC [2,7].

Consequently, the demand for minimally invasive scarless treatment has been increasing; thus, ethanol ablation (EA) has been gaining popularity as an optimal alternative for the treatment of TGDC, being associated with high feasibility, a high safety profile, and favorable treatment outcomes [7,11,12,13,14,15,16]. However, to our best knowledge, only seven studies on the use of EA as a primary treatment option for TGDC have been published since 2011; thus, there is a lack of consensus on several issues related to EA for TGDC, particularly on its detailed techniques and role as a primary treatment for TGDC. A systematic review and meta-analysis on chemical ablation using ethanol or OK-432 for treating TGDC was published in 2017. Nevertheless, the article only included four studies on EA for TGDC, primarily focusing on treatment outcomes with minimal discussion about the current issues with EA [11].

This article aims to provide a comprehensive review involving seven studies on EA for TGDC published since 2011 (Table 1), addressing several technical issues of EA and its possible role as a new standard of treatment for TGDC.

## 2. Overall Practice of EA for TGDC

### 2.1. Pre-Procedural Evaluation

A benign TGDC must be accurately diagnosed before EA, thereby differentiating it from dermoid or lymph nodes. Moreover, possible occult malignancy from TGDC should also be considered, even though it is an extremely rare condition with an incidence rate of approximately 1–2% [6,17]. Ultrasonography (US) and computed tomography scans are the commonly used imaging modalities for evaluating the characteristics of TGDC. Additionally, US-guided fine-needle aspiration cytology (US-FNAC) is routinely performed to diagnose benign TGDC and rule out malignancy. In some patients, the measurement of thyroglobulin levels in the washout fluid of the FNAC needle could be helpful in differentiating thyroid follicle-containing lesions (benign TGDC or TGDC carcinoma) from other lesions (dermoid or lymph node).

**Figure 1:**
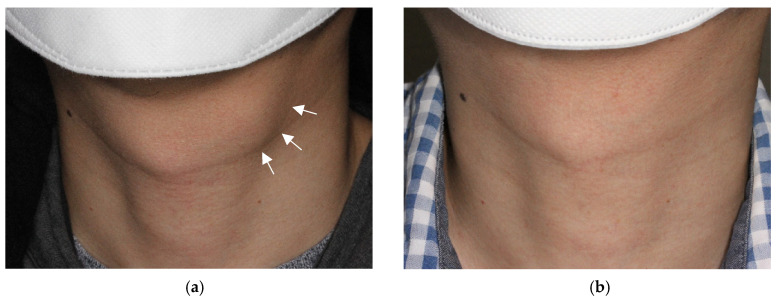
Photograph of an external neck with a thyroglossal duct cyst. The mass (arrows) is easily visible in the off-midline anterior neck at the hyoid level, being classified as World Health Organization (WHO) cosmetic score 4 (**a**). After ethanol ablation, the mass is no longer identified on physical examination, with a classification of WHO cosmetic score 1 (**b**).

The baseline volume of TGDC is determined by measuring the diameters of the largest axis (a) and the two remaining perpendicular axes (b, c) on US. Using the formula for measuring the volume of an elliptical sphere (V = πabc/6), the volume is calculated. The volume reduction rate (VRR) is calculated as follows: VRR = (initial volume − final volume)/initial volume × 100% after treatment. The cosmetic deformity caused by TGDC is assessed using the World Health Organization (WHO) cosmetic scoring system (1, no palpable lesion; 2, with palpable lesion but no cosmetic problem; 3, lesion visible only to an experienced physician; and 4, easily visible mass) (Figure 1).

### 2.2. Aspiration of Internal Contents and Ethanol Injection

**Figure 2:**
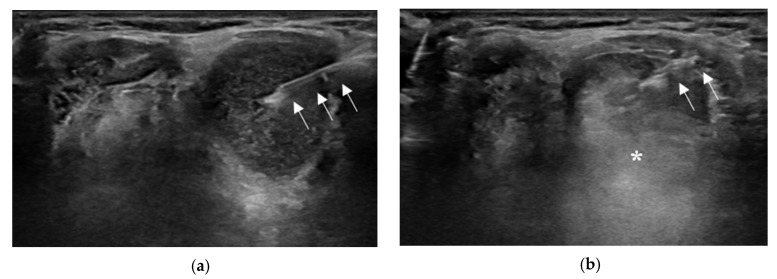
Ultrasound (US)-guided ethanol ablation procedure. The needle (arrows) is inserted into the cyst under real-time US monitoring (**a**). After cystic fluid aspiration, 99% sterile ethanol is injected into the cystic cavity, which appears as a hyperechoic flush (asterisk) on real-time US (**b**).

The US-EA technique is quite simple, and the procedure is generally performed in an outpatient setting. Patients are placed in a supine position with their necks extended. After the needle path is planned with the US examination, an alcohol swab is used to sterilize the skin, similar to the FNAC procedure. Local anesthesia is not mandatory because the procedure is usually completed with a single needle puncture. After the needle is inserted into the cyst under real-time US monitoring, the cystic fluid is aspirated as much as possible. Subsequently, 99% sterile ethanol is injected into the cystic cavity (Figure 2).

### 2.3. Technical Issues or Variations in EA for TGDC

#### 2.3.1. Local Anesthesia

Of the seven studies published since 2011, only the two studies by Lee et al. and Park et al. stated that local anesthesia was performed by injecting lidocaine [14,16]. The two studies by Chow et al. and Ahn et al. clearly stated that local anesthesia was not used, while the remaining three studies did not include any description regarding local anesthesia use [7,12,13,15,18].

#### 2.3.2. Needle Size Used for EA

In EA for TGDC, 18–23-gauge needles are used, and various needle sizes can be used in a single procedure [7,11,12,13,15,16,18]. An 18-gauge needle was used in the two studies by Lee et al. and Karatay et al., while an 18- or 21-gauge needle was used in the two studies by Chung et al. and Park et al. [12,14]. In the studies by Kim et al., Chow et al., and Ahn et al., 21-, 20–22-, and 21–23-gauge needles were used, respectively [7,13,18]. In several studies, 14- or 16-gauge large-bore needles were also used to facilitate the complete aspiration of the cystic fluid if the internal contents of the TGDC were highly viscous [13,15,16].

#### 2.3.3. Amount of Ethanol Injected

Although the appropriate amount of ethanol injection has not been investigated in studies on EA for TGDC, approximately ≥50% of the aspirate volume with ≤10 mL of ethanol is usually used, similar to the EA protocol for cystic thyroid nodules [11,13,14,15,18]. Among the seven studies on EA for TGDC, the five studies by Kim et al., Chow et al., Lee et al., Chung et al., and Park et al. used 50% or more of the aspirate volume as the lowest limit of ethanol injection, using up to 15 mL of ethanol [13,14,15,16,18]. However, the recent two studies by Karatay et al. and Ahn et al. used a relatively small volume of ethanol, which was ≤5 mL and ≤2.5 mL, respectively [7,12].

#### 2.3.4. Retention or Aspiration of Injected Ethanol

Among the seven studies on EA for TGDC published since 2011, six studies explored whether the authors used retention or aspiration of the injected ethanol [7,12,13,14,15,16]. The four studies by Kim et al., Lee et al., Chung et al., and Park et al. used an aspiration method that involved aspiration of the injected ethanol at the end of the procedure with 5–10 min of retention time [13,14,15,16]. Conversely, the recent two studies by Karatay et al. and Ahn et al. did not aspirate the injected ethanol and used retention methods [5,12].

#### 2.3.5. EA for TGDC with Viscous Internal Contents

A critical factor for treatment success in EA of a benign cystic lesion is the sufficient evacuation of the internal cystic fluid. Thus, a cystic mass with viscous internal contents could provide therapeutic challenges in EA. Although the literature elucidating specifically on EA for viscous TGDC is lacking, several techniques have been used for the successful evacuation of viscous cystic fluid based on the studies on EA for viscous thyroid cyst, which include normal saline irrigation to decrease viscosity, repeated ethanol injection with an interval period to liquefy the viscous contents, use of a large-bore needle or catheter, and use of a suction pump [13,15,16]. In the studies on EA for TGDC by Kim et al., Chung et al., and Park et al., large-bore needles (14- or 16-gauge) with connection to a large-volume syringe (20 mL or 50 mL) were used if the internal contents were not aspirated due to high viscosity [13,15,16]. Kim et al. described that a suction pump was also used to facilitate complete aspiration of the internal contents [13]. To remove internal debris or viscous materials within the cyst, Lee et al. and Park et al. performed irrigation with lidocaine or normal saline [14,16].

#### 2.3.6. Number of Treatment Sessions

In the studies on EA for TGDC, from one to six treatment sessions were reported [7,12,13,14,15,16,18]. The mean number of treatment sessions ranged from 1 to 1.7 in seven different studies, and multiple treatments were provided in approximately 0.0–44.4% of patients [7,12,13,14,15,16,18]. In the study by Kim et al., involving 11 patients with TGDC in 2011, repeat EA was performed in approximately 27% of patients [13]. According to the study by Chung et al., the treatment success rate (TSR) after the first session of treatment was 92.7%; however, 26.8% (15/56) of patients ultimately received multiple treatment sessions due to various reasons such as no improvement in symptoms or signs, insufficient VRR (<50%), and regrowth of the lesion during the follow-up [15]. In the study by Ahn et al., the cumulative TSRs after the first, second, and third sessions of EA were 82.1% (23/28), 96.4% (27/28), and 96.4% (27/28), respectively [7]. Therefore, we assumed that at least 17.9% (5/28) of patients might require two or more treatment sessions to achieve treatment success.

## 3. EA Results for TGDC

### 3.1. Treatment Efficacy

The treatment efficacy after EA is usually assessed using two parameters, that is, VRR and TSR. The VRR is an objective value of the volume decrease in TGDC calculated by the following formula: (initial volume − final volume)/initial volume × 100%; the TSR is a semi-objective value defined by physicians based on the VRR, and 50% or 70% of the VRR was commonly used as cutoff values for TSR in previous studies [7,13,14,15,16,18,19].

Among the seven studies published since 2011, six studies reported their treatment outcomes using VRR, and the mean or median VRR at the last follow-up was 76.6–96.4% in different studies [7,12,13,14,15,16]. Although the definition of TSR was different among the studies, TSR was estimated in five studies, and it ranged from 77.8% to 96.4% [7,13,14,15,16]. In reviewing studies involving more than 20 patients, Chung et al. reported an 82.3% VRR and 80.4% TSR when defined as VRR ≥50% at the 10-month follow-up in 56 patients [15]. Karatay et al. reported a 95.1% VRR at the 12-month follow-up in 28 patients [12]. Park et al. reported a 73% VRR at the 3-month follow-up and an 81% VRR at the last follow-up in 68 patients [16]. The TSR was 83% in the study when it was defined as VRR ≥ 50%. Most recently, Ahn et al. reported a 96.2% VRR and 96.4% TSR (VRR ≥ 70%) at the last follow-up with 22 months of the median follow-up period [7]. In a recent systematic review article on the chemical ablation of TGDC involving 82 patients with EA from four studies, the pooled success rate calculated using the random effects model was 84% (95% CI: 74–90%) in the ethanol group [11].

### 3.2. Outcome-Related Factors

Outcome-related factors were only evaluated in one study [7]. In that study, the authors assessed the treatment outcomes of EA, including the VRR, TSR, and number of treatment sessions, according to TGDC characteristics such as the initial volume, presence of septation, presence of debris, and viscosity of the cyst fluid (low: watery; intermediate: creamy; high: sticky or gelatinous). However, they did not find any between-group differences in the VRR, treatment success, or number of treatment sessions as functions of TGDC characteristics. This might be primarily attributed to the small number of cases in each subgroup. However, in contrast, this finding may indicate that EA could provide consistent treatment efficacy regardless of the various characteristics of TGDC.

### 3.3. Complications

In the literature on EA for benign thyroid nodules, major complications such as dysphonia and infection with/without a thyroid abscess have been reported [20,21,22]. Regarding EA for TGDC, only one case of wound infection treated with surgery has been reported [16]. No other major complications have been reported in a total of 206 patients from the seven studies included in this review, demonstrating a high safety profile for EA in patients with TGDC [7,11,12,13,14,15,16,18]. In a recent systematic review that evaluated the outcomes of chemical ablation for TGDC in 82 patients with EA from four studies, major complications were not identified [11]. Although it would not be a true complication, pain has been reported as the most common adverse presentation associated with EA. The studies by Kim et al., Chow et al., and Karatay et al. reported pain as the only complication of EA [12,13,18]. According to a recent prospective case series published in 2023, 92.9% (26/28) of patients experienced procedure-related pain, particularly during needle withdrawal after ethanol injection [7]. However, the pain was usually mild and lasted for a short time. Therefore, a high incidence of tolerable pain was not a critical consideration when adopting EA in patients with TGDC [7]. Other than pain, Chung et al. reported a temporary inspiratory stridor in one patient (1.8%, 1/56) without hypoxia who was treated with conservative treatment [15].

### 3.4. Cost

It is easily presumed that the cost of EA should be lower than that of surgery because EA is usually performed in an office-based setting without general anesthesia. However, data remain lacking regarding the cost or cost-effectiveness of EA for TGDC. Thus far, only one study published in 2017 addressed the cost of EA in comparison with surgery [15]. In the study, the median cost of EA was approximately USD 326 (range USD 200−384), while the cost of surgery was approximately USD 1104 (range USD 599−2807), with acceptable treatment efficacy in both modalities (treatment failure rate was 19.6% in the EA group and 2.4% in the surgery group) [15]. Therefore, EA might be cost-effective as a primary treatment for TGDC. However, when assessing the cost or cost-effectiveness of EA for TGDC, we should consider the fact that an additional cost is incurred through regular follow-up US examinations to evaluate the treatment results after EA, and some patients require multiple treatment sessions to achieve treatment success, leading to an increase in the total cost of EA.

## 4. Discussion

EA is widely used as a non-surgical, minimally invasive treatment for various disease entities. Cystic thyroid nodules are the most common candidates for EA use, and the clinical practice and detailed technique have been well established through several international guidelines and consensus statements [23,24,25,26]. Moreover, treatment efficacy and EA complications for thyroid nodules have also been comprehensively understood based on several review articles and meta-analyses [20,21]. Other than cystic thyroid nodules, various benign cystic lesions of the head and neck, including TGDC, branchial cleft cyst, and ranula, are also candidates for EA use [19,27,28]. TGDC is the second most common pathology for EA use next to cystic thyroid nodules, as it is the most common congenital neck mass frequently encountered in clinical practice [2,9]. In contrast to EA for cystic thyroid nodules, however, established techniques or guidelines for EA for TGDC are lacking, requiring a comprehensive review of the current practice of EA for TGDC.

### 4.1. Needs for Local Anesthesia

Because TGDC does not involve sensory innervation and the procedure is completed with a single needle puncture, EA for TGDC could be performed in an outpatient setting even without local anesthesia. Although Ahn et al. reported that most patients (92.9%) experienced mild-to-moderate pain during withdrawal of the inserted needle, the small amount of ethanol leakage through the needle puncture site is inevitable during needle withdrawal following ethanol injection, causing mild pain [7]. Therefore, such pain could not be prevented by local anesthesia. Moreover, pain should not be prevented because the development of unexpected pain during ethanol injection is an important sign of inadvertent ethanol seepage, which may lead to surrounding tissue damage. However, in cases of huge TGDC or TGDC with highly viscous internal contents, which may require multiple aspirations or irrigations, local anesthesia may reduce the patient’s discomfort induced by the use of a large-bore needle or multiple needle punctures. The administration of 1–2% lidocaine with or without 1:100,000 epinephrine is the most commonly used local anesthetic method. However, topical anesthetic cream can also be beneficial as a topical anesthetic agent by preventing the additional pain caused by the lidocaine injection.

### 4.2. Needle Size Used for EA

Various needle sizes (18–23-gauge) have been used for EA in the literature [7,12,13,14,15,16,18]. No rule or consensus exists regarding which needle size is the most optimal for EA in TGDC. The needle size should be determined and individualized based on the TGDC characteristics, particularly the volume and viscosity of the internal contents. A larger-bore needle is generally preferred for TGDCs with large volumes or viscous internal contents to facilitate effective aspiration of the cystic fluid. However, the operator should consider that the use of a larger-bore needle is possibly associated with a larger seepage of injected ethanol at the time of needle withdrawal, and this might lead to more procedure-related pain.

### 4.3. Amount of Ethanol Injected

The available literature and evidence discussing the appropriate amount of ethanol in EA for TGDC are limited. In 71.4% (5/7) of the studies published since 2011, at least 50% of the aspirate volume was used as the volume of the ethanol injection. Moreover, an amount of ethanol up to 90% of the aspirate volume was also reported. Indeed, there is no consensus on the appropriate amount of ethanol in EA for TGDC; thus, most studies on EA for TGDC adopted an EA protocol for cystic thyroid nodules, where the technique or practice of EA is well-established. However, even in EA for cystic thyroid nodules, the scientific background or rationale for using an ethanol volume of ≥50% of the aspirate volume has not been investigated. In fact, a recent systematic review of EA for cystic thyroid nodules involving 19 studies reported that a lower volume of ethanol injection (<50% of the initial cyst volume) was significantly associated with a higher VRR [20]. Additionally, a study by Cho et al. in 2021 also demonstrated a high TSR (88.5%) even after a single session of EA using low-dose ethanol (≤5 mL) [29]. The study suggested that EA using low-dose ethanol is also effective for the treatment of symptomatic cystic thyroid nodules, regardless of the initial cyst volume and properties of the aspirate [29]. In the most recent study on EA for TGDC by Ahn et al., although the mean volume of the ethanol injected during EA was only 33.3% of the aspirate, the VRR and TSR were 96.2% and 96.4%, respectively, which were relatively higher than those reported in previous studies using an ethanol volume of ≥50% of the aspirate [7]. Therefore, 50% of the aspirate volume as the lower limit of the ethanol injection volume is not the tenet in terms of treatment efficacy. Considering that the treatment effect of EA occurred through cell dehydration and protein denaturation of secretory lining cells of the cystic wall by ethanol exposure to these cells, the effective ethanol volume would not depend on the initial cyst volume or aspiration volume but on the volume of the collapsed cyst following aspiration [7,23]. Therefore, further studies should be conducted to find the best effective dose of ethanol in EA for TGDC without compromising its feasibility and safety profile.

### 4.4. Retention or Aspiration of Injected Ethanol

Although four out of the seven studies used aspiration methods (aspiration of injected ethanol after a short retention time), there is no consensus on whether ethanol should be fully aspirated after instillation [13,14,15,16,24]. In the aspiration method, the retention time was 5–10 min [13,14,15,16]. Referring to the study of EA for benign thyroid cysts, aspiration of the injected ethanol is preferred, based on the theoretical rationale that the retention of the injected ethanol may increase patient inconvenience and cause complications due to possible ethanol leakage [23,30,31]. The studies on EA for benign thyroid cysts by Kim et al. and Park et al. showed no difference in VRR or complications in the groups in which ethanol was retained or aspirated after injection, while other studies have suggested that the complete removal of ethanol after a short retention time decreases the rates of ethanol leakage and may increase patient satisfaction after the procedure [24,30,31]. However, injected ethanol retention may be associated with better treatment outcomes because the duration of the chemical reaction would be much longer than with the aspiration method. In the study by Park et al., a trend toward a higher TSR in the retention group, particularly in cases of predominantly cystic lesions rather than pure cystic lesions, was observed [30]. Furthermore, a recent systematic review of EA for cystic thyroid nodules demonstrated that studies using the retention technique rather than the aspiration technique had significantly higher VRRs than other non-surgical options [20]. In the current review of EA for TGDC, the VRR and TSR of the two studies using the retention method were relatively higher compared with those of the four studies using aspiration methods, although the statistical analysis was not available (95.1–96.2% of VRR and 96.4% of TSR in the two studies using the retention method vs. 76.6–82.3% of VRR and 77.8–83% of TSR in the four studies using the aspiration method) [7,12,13,14,15,16]. Additionally, we could not identify any differences in the complication rates between the retention and aspiration methods both in EA for benign thyroid cysts and TGDC, as EA is highly safe, regardless of this minor technique variation. However, regarding EA for TGDC, studies directly comparing the treatment efficacy and complications according to the retention and aspiration methods are lacking. Therefore, this technical issue is still left to the operator’s discretion [24].

### 4.5. Number of Treatment Sessions

In contrast to surgery, EA may be associated with multiple courses of treatment. In this review, the mean number of EA sessions ranged from 1 to 1.7. Of the 206 patients from the seven studies, 41 patients (19.9%) received multiple sessions of EA for various reasons, including failure to achieve treatment success after the first session of EA, regrowth of the mass during follow-up, and prevention of possible recurrence [7,12,13,14,15,16,18]. However, no study has revealed which factors are associated with the need for multiple sessions of EA to achieve treatment success, and those may involve several patient/TGDC characteristics such as age, sex, history of infection, initial volume, septation, debris, and cyst fluid viscosity. Therefore, this issue should be addressed in future studies to identify risk factors for requiring multiple treatment sessions and to predict the TSR of EA as a single-stage treatment. Moreover, physicians should provide comprehensive information regarding the possible need for multiple treatment sessions and the lengthening of the overall treatment course when adopting EA as a primary treatment modality for TGDC.

### 4.6. Potential of EA as a Primary Treatment of TGDC in Terms of Feasibility, Safety, and Treatment Efficacy

EA is an office-based, minimally invasive technique that does not require anesthesia. Additionally, according to a recent study by Ahn et al., all procedures are completed within 10 min in an outpatient setting without hospitalization [7]. Conversely, the Sistrunk operation is usually performed under general anesthesia and requires 1–2 h of surgical time. Therefore, EA may provide a higher degree of feasibility for TGDC treatment compared with the Sistrunk operation for both patients and surgeons, being less restricted by the patient’s comorbidities, treatment time, and treatment place. In terms of safety, the overall complication rate after EA is <1%, and no major complications have been reported, while the Sistrunk operation has been associated with 7.5–33% of the overall complication rate [10,32,33,34]. Regarding treatment efficacy, EA demonstrated a 76.6–96.2% VRR and 77.8–96.4% TSR in different studies [7,11,12,13,14,15,16]. Given that the recurrence rate of a Sistrunk operation has been reported to be 1.9–27.3%, the treatment efficacy of EA would be comparable with that of the Sistrunk operation [2,9,10,32,33,34]. With this evidence for feasibility, safety, and treatment efficacy, EA could be suggested as a primary treatment modality in patients with TGDC instead of the Sistrunk operation. However, only one study, published by Chung et al. in 2017, directly compared the treatment outcomes of EA with surgery. In the study, which involved 56 and 289 patients in the EA and surgery groups, respectively, the authors concluded that EA had an acceptable treatment efficacy with a better safety profile than surgery; however, the treatment failure rate of EA was higher than that of surgery (19.6% vs. 2.4%, respectively, *p* < 0.001). Furthermore, there is a possible concern regarding the short duration of the therapeutic effect of EA or the early regrowth of ablated TGDC after EA because EA is not a treatment that completely eradicates the mass, unlike surgery. Although a recent study by Park et al. in 2022 reported that TSR was as high as 83% (35/45) even in patients who were followed for more than 2 years, the study had a selection bias, and the clinical evidence to guarantee the long-term success of EA in patients with TGDC is lacking [16]. Moreover, the indications and patient selection for EA could differ from those for surgery because EA is not always available for all patients with TGDC, particularly complex and/or pediatric TGDCs. Therefore, when comparing the treatment efficacy or complications between EA and surgery, these aspects should be considered. Despite the bias in patient selection and the relatively insufficient clinical data compared with the Sistrunk procedure, it is clear that EA is a strongly attractive option in the management of benign TGDC that allows patients to avoid various surgical burdens and provides favorable treatment outcomes without complications.

### 4.7. Practical Limitations of EA for TGDC

EA is challenging to use for complicated TGDCs, particularly TGDCs with a cutaneous fistula, because the retention of injected ethanol within the cyst is not feasible. Additionally, this technique may require general anesthesia for pediatric patients who cannot cooperate during the procedure. Given the fact that approximately 50% of TGDCs manifest in pediatric patients, additional studies involving pediatric patients should be performed for EA to be recognized as a true primary treatment for all patients with TGDC [2,9].

## 5. Study Limitations

This review had several strengths and limitations. All published research studies on EA for TGDC were comprehensively reviewed with in-depth discussions about the current issues of EA. Although this review provided the most updated data from the studies included, new information from additional meta-analyses could not be presented because the total number of studies was still small and there was a significant heterogeneity in the detailed EA techniques among the studies, along with different definitions of TSR in each study.

## 6. Conclusions and Future Directions

EA is a highly feasible treatment for TGDC that can be performed in an outpatient setting without surgical burdens such as general anesthesia and surgical scars. Furthermore, this technique has a high safety profile without major complications and can provide favorable treatment efficacy. Based on the current evidence, EA can be a primary treatment modality for patients with TGDC. However, several issues on the technical aspect, such as the amount of ethanol injection and the aspiration/retention of injected ethanol, should be discussed more in future studies to establish a consistent practice of EA for TGDC. With respect to outcomes, effort should be exerted to explore outcome-related factors through larger-scale studies to stratify the risk of treatment failure and predict EA outcomes. Additionally, there is a demand for in vitro and randomized, controlled clinical studies to evaluate the long-term success of EA. 

## Figures and Tables

**Table 1:** Summary of seven studies on ethanol ablation for thyroglossal duct cyst since 2011.

Author (Publication Year)	Study Design	Study Period	Patient Number	Age (Years)	Male:Female	Initial Volume (mL)	Local Anesthesia	Needle	Aspirate Volume (mL)	Injection Amount
Kim [13] (2011)	RCS	2005–2008	11	34.9 (23–44)	3:08	6.0 (0.7–29.4)	N/A	21	4.6 (0.5–25)	50–80% of aspirated volume
Chow [17] (2012)	RCS	NA	6	44.8 (37–61)	2:04	4.9 (1.5–9.8)	No	20–22	N/A	50–90% of aspirated volume
Lee [14] (2015)	RCS	2012–2013	9	36 (14–58)	7:02	8.9 (0.2–36.9)	Yes	18	N/A	70–80% of cystic volume
Chung [15] (2017)	RCC	2005–2014	56	37.5 ^a^ (14–71)	23:33	3.9 ^a^ (0.3–26.6)	N/A	18 or 21	4.4 (0–25)	50% of aspirated volume
Karatay [12] (2021)	RCS	2018–2020	28	42 ^a^ (19–0–76)	14:14	4.1 ^a^ (1.0–15.1)	N/A	18	N/A	≤5 mL
Park [16] (2022)	RCS	2008–2018	68	38 ± 16	21:47	7.3 ± 19.0	Yes	18 or 21	N/A	50% of aspirated volume
Ahn [7] (2023)	RCS	2016–2021	28	47 (20–69)	16:12	6.7 ^a^ (1.4–20.1)	No	21–23	5.0 ^a^ (0–18)	≤2.5 mL
**Author (Publication** ** Year)**	**Number of EA Sessions**	**Multiple EA Sessions**	**Aspiration of Injected Ethanol**	**Retention Time (min)**	**Volume at Last Follow-Up (mL)**	**VRR at Last Follow-Up**	**TSR**	**Follow-Up (Months)**	**Complications**	
Kim [13] (2011)	1.4 (1–3)	3 (27%)	Yes	10	0.36 (0–1.08)	81.3% (43.9–98.3%)	80% ^b^	13.6 (3–29)	Mild pain	
Chow [17] (2012)	1.3 (1–3)	1 (16.8%)	N/A	N/A	N/A	N/A	N/A (relapse in one patient)	34.5 (17–72)	Moderate pain in two patients	
Lee [14] (2015)	1.7 (1–3)	4 (44.4%)	Yes	5	1.9	76.6% (−4–100%)	77.8% ^c^	13.1 (12–15)	No	
Chung [15] (2017)	1.5 (1–6)	15 (26.8%)	Yes	10	0.8 ^a^ (0–14.9)	82.30%	80.4% ^b^	10 ^a^ (1–94)	Temporary inspiratory stridor in one patient	
Karatay [12] (2021)	1	0 (0.0%)	No	N/A	0.01 ^a^ (0–1.2)	95.10%	N/A	12	Temporary pain in three patients	
Park [16] (2022)	1.1 (1–3)	8 (11.2%)	Yes	10	N/A	81%	Immediate, 81% ^b^; long-term, 83% ^b^	69 ^a^ (24–131) for 42 patients	Wound infection in one patient	
Ahn [7] (2023)	1.4 (1–3)	10 (35.7%)	No	N/A	0.2 ^a^ (0.2–2.8)	96.2% ^a^ (58.1–100)	96.4%^c^	22 ^a^ (9–65)	Mild-to-moderate pain	

RCS, retrospective case series; RCC, retrospective case-control; continuous variables are presented as mean (range) if range was provided in the study; EA, ethanol ablation; VRR, volume reduction rate; TSR, treatment success rate; continuous variables are presented as mean (range) if range was provided in the study; ^a^ median; ^b^ TSR was defined as a VRR of ≥50%; ^c^ TSR was defined as a VRR of ≥ 70%.

## References

[1] Flint P.W., Haughey B.H., Robbins K.T., Thomas J.R., Niparko J.K., Lund V.J., Lesperance M.M. (2014). Cummings Otolaryngology-Head and Neck Surgery E-Book.

[2] Gioacchini F.M., Alicandri-Ciufelli M., Kaleci S., Magliulo G., Presutti L., Re M. (2015). Clinical presentation and treatment outcomes of thyroglossal duct cysts: A systematic review. Int. J. Oral Maxillofac. Surg..

[3] Galluzzi F., Pignataro L., Gaini R.M., Hartley B., Garavello W. (2013). Risk of recurrence in children operated for thyroglossal duct cysts: A systematic review. J. Pediatr. Surg..

[4] Goldsztein H., Khan A., Pereira K.D. (2009). Thyroglossal duct cyst excision—The Sistrunk procedure. Oper. Tech. Otolaryngol.-Head. Neck Surg..

[5] Shah R., Gow K., Sobol S.E. (2007). Outcome of thyroglossal duct cyst excision is independent of presenting age or symptomatology. Int. J. Pediatr. Otorhinolaryngol..

[6] Thompson L.D.R., Herrera H.B., Lau S.K. (2017). Thyroglossal Duct Cyst Carcinomas: A Clinicopathologic Series of 22 Cases with Staging Recommendations. Head. Neck Pathol..

[7] Ahn D., Kwak J.H., Lee G.J., Sohn J.H. (2023). Ultrasound-Guided Ethanol Ablation as a Primary Treatment for Thyroglossal Duct Cyst: Feasibility, Characteristics, and Outcomes. Otolaryngol. Head. Neck Surg..

[8] Rayess H.M., Monk I., Svider P.F., Gupta A., Raza S.N., Lin H.-S. (2017). Thyroglossal duct cyst carcinoma: A systematic review of clinical features and outcomes. Otolaryngol.–Head. Neck Surg..

[9] Malka Yosef L., Lahav Y., Hazout C., Zloczower E., Halperin D., Cohen O. (2021). Impact of age on surgical outcomes and failure rates in patients with thyroglossal duct cysts. Am. J. Otolaryngol..

[10] Ross J., Manteghi A., Rethy K., Ding J., Chennupati S.K. (2017). Thyroglossal duct cyst surgery: A ten-year single institution experience. Int. J. Pediatr. Otorhinolaryngol..

[11] Park S.I., Baek J.H., Suh C.H., Chung S.R., Choi Y.J., Kim T.Y., Lee Y.-M., Lee J.H. (2021). Chemical ablation using ethanol or OK-432 for the treatment of thyroglossal duct cysts: A systematic review and meta-analysis. Eur. Radiol..

[12] Karatay E., Javadov M. (2021). The effectiveness of ethanol ablation in the treatment of thyroglossal duct cysts in adult cases and evaluation with cosmetic scoring. Jpn. J. Radiol..

[13] Kim S.M., Baek J.H., Kim Y.S., Sung J.Y., Lim H.K., Choi H., Lee J. (2011). Efficacy and safety of ethanol ablation for thyroglossal duct cysts. Am. J. Neuroradiol..

[14] Lee D.K., Seo J.W., Park H.S., Kang M.K., Jang A.L., Lee J.H., Hong J.C. (2015). Efficacy of ethanol ablation for thyroglossal duct cyst. Ann. Otol. Rhinol. Laryngol..

[15] Chung M.S., Baek J.H., Lee J.H., Choi Y.J., Yoon J.H., Nam S.Y., Kim S.C., Sung J.Y., Baek S.M., Na D.G. (2017). Treatment Efficacy and Safety of Ethanol Ablation for Thyroglossal Duct Cysts: A Comparison with Surgery. Eur. Radiol..

[16] Park S.I., Baek J.H., Chung S.R., Choi Y.J., Lee J.H., Kim T.Y., Lee Y.M., Baek S.M. (2022). Ethanol ablation for the treatment of thyroglossal duct cysts: Follow-up results for longer than 2 years. Eur. Radiol..

[17] Aculate N.R., Jones H.B., Bansal A., Ho M.W. (2014). Papillary carcinoma within a thyroglossal duct cyst: Significance of a central solid component on ultrasound imaging. Br. J. Oral Maxillofac. Surg..

[18] Chow T.L., Choi C.Y., Yee-Hing Hui J. (2012). Thyroglossal duct cysts in adults treated by ethanol sclerotherapy: A pilot study of a nonsurgical technique. Laryngoscope.

[19] Lee E., Park I., Elzomor A., Li L., Lloyd A., Benito D.A., Goodman J.F., Thakkar P.G., Joshi A. (2021). Efficacy of ethanol ablation as a treatment of benign head and neck cystic lesions. Am. J. Otolaryngol..

[20] Yang C.C., Hsu Y., Liou J.Y. (2021). Efficacy of Ethanol Ablation for Benign Thyroid Cysts and Predominantly Cystic Nodules: A Systematic Review and Meta-Analysis. Endocrinol. Metab..

[21] Cesareo R., Tabacco G., Naciu A.M., Crescenzi A., Bernardi S., Romanelli F., Deandrea M., Trimboli P., Palermo A., Castellana M. (2022). Long-term efficacy and safety of percutaneous ethanol injection (PEI) in cystic thyroid nodules: A systematic review and meta-analysis. Clin. Endocrinol..

[22] Mulita F., Tchabashvili L., Verras G.I., Liolis E., Siouti S., Panagopoulos K., Vailas M. (2021). Thyroid abscess as a complication of percutaneous ethanol ablation of cystic thyroid nodules. Endokrynol. Pol..

[23] Hahn S.Y., Shin J.H., Na D.G., Ha E.J., Ahn H.S., Lim H.K., Lee J.H., Park J.S., Kim J.H., Sung J.Y. (2019). Ethanol Ablation of the Thyroid Nodules: 2018 Consensus Statement by the Korean Society of Thyroid Radiology. Korean J. Radiol..

[24] Orloff L.A., Noel J.E., Stack B.C., Russell M.D., Angelos P., Baek J.H., Brumund K.T., Chiang F.Y., Cunnane M.B., Davies L. (2022). Radiofrequency ablation and related ultrasound-guided ablation technologies for treatment of benign and malignant thyroid disease: An international multidisciplinary consensus statement of the American Head and Neck Society Endocrine Surgery Section with the Asia Pacific Society of Thyroid Surgery, Associazione Medici Endocrinologi, British Association of Endocrine and Thyroid Surgeons, European Thyroid Association, Italian Society of Endocrine Surgery Units, Korean Society of Thyroid Radiology, Latin American Thyroid Society, and Thyroid Nodules Therapies Association. Head Neck.

[25] Papini E., Monpeyssen H., Frasoldati A., Hegedus L. (2020). 2020 European Thyroid Association Clinical Practice Guideline for the Use of Image-Guided Ablation in Benign Thyroid Nodules. Eur. Thyroid J..

[26] Feldkamp J., Grunwald F., Luster M., Lorenz K., Vorlander C., Fuhrer D. (2020). Non-Surgical and Non-Radioiodine Techniques for Ablation of Benign Thyroid Nodules: Consensus Statement and Recommendation. Exp. Clin. Endocrinol. Diabetes.

[27] Ryu K.H., Lee J.H., Lee J.Y., Chung S.R., Chung M.S., Kim H.W., Choi Y.J., Baek J.H. (2017). Ethanol Ablation of Ranulas: Short-Term Follow-Up Results and Clinicoradiologic Factors for Successful Outcome. Am. J. Neuroradiol..

[28] Ha E.J., Baek S.M., Baek J.H., Shin S.Y., Han M., Kim C.H. (2017). Efficacy and Safety of Ethanol Ablation for Branchial Cleft Cysts. Am. J. Neuroradiol..

[29] Cho W., Sim J.S., Jung S.L. (2021). Ultrasound-guided ethanol ablation for cystic thyroid nodules: Effectiveness of small amounts of ethanol in a single session. Ultrasonography.

[30] Park H.S., Yim Y., Baek J.H., Choi Y.J., Shong Y.K., Lee J.H. (2019). Ethanol ablation as a treatment strategy for benign cystic thyroid nodules: A comparison of the ethanol retention and aspiration techniques. Ultrasonography.

[31] Kim D.W., Rho M.H., Kim H.J., Kwon J.S., Sung Y.S., Lee S.W. (2005). Percutaneous ethanol injection for benign cystic thyroid nodules: Is aspiration of ethanol-mixed fluid advantageous?. Am. J. Neuroradiol..

[32] Hirshoren N., Neuman T., Udassin R., Elidan J., Weinberger J.M. (2009). The imperative of the Sistrunk operation: Review of 160 thyroglossal tract remnant operations. Otolaryngol. Head Neck Surg..

[33] Maddalozzo J., Venkatesan T.K., Gupta P. (2001). Complications associated with the Sistrunk procedure. Laryngoscope.

[34] Lekkerkerker I., van Heurn E.L., van der Steeg A.F., Derikx J.P. (2019). Pediatric thyroglossal duct cysts: Post-operative complications. Int. J. Pediatr. Otorhinolaryngol..

